# Subtype Characterization of Ovarian Cancer Cell Lines Using Machine Learning and Network Analysis: A Pilot Study

**DOI:** 10.3390/cancers17213509

**Published:** 2025-10-31

**Authors:** Rama Krishna Thelagathoti, Dinesh S. Chandel, Chao Jiang, Wesley A. Tom, Gary Krzyzanowski, Appolinaire Olou, M. Rohan Fernando

**Affiliations:** Molecular Diagnostic Research Laboratory, Center for Sensory Neuroscience, Boys Town National Research Hospital, Omaha, NE 68131, USA; ramakrishna.thelagathoti@boystown.org (R.K.T.); chao.jiang@boystown.org (C.J.); appolinaire.olou@boystown.org (A.O.)

**Keywords:** cancer informatics, dimensionality reduction, machine learning, transcriptome profiling, network analysis, cancer biomarkers

## Abstract

**Simple Summary:**

Ovarian cancer is a heterogeneous disease with multiple molecular subtypes that influence treatment response and prognosis. Identifying these subtypes from high-dimensional genomic data remains challenging due to the complexity and noise inherent in transcriptomic datasets. In this pilot study, we developed a multi-stage computational framework to reduce dimensionality and uncover biologically meaningful subgroups. Starting from over 63,000 mRNA features, variance-based filtering, correlation pruning, and supervised selection narrowed the dataset to 80 highly discriminative transcripts. Using these features, we constructed co-expression similarity networks, and visual inspection of the network topology revealed four distinct groups among seven ovarian cancer cell lines. These subtypes aligned with known biological and mutational profiles including TP53-driven high-grade serous groups, PI3K/AKT- and ARID1A-associated clear cell/endometrioid groups, a drug-resistant OVCAR-3 group, and a hybrid IGROV-1 profile. This integrative approach demonstrates that combining feature selection with network modeling provides a powerful method for resolving ovarian cancer heterogeneity and improving subtype-specific insights.

**Abstract:**

**Background/Objectives:** Ovarian cancer is a heterogeneous malignancy with molecular subtypes that strongly influence prognosis and therapy. High-dimensional mRNA data can capture this biological diversity, but its complexity and noise limit robust subtype characterization. Furthermore, current classification approaches often fail to reflect subtype-specific transcriptional programs, underscoring the need for computational strategies that reduce dimensionality and identify discriminative molecular features. **Methods:** We designed a multi-stage feature selection and network analysis framework tailored for high-dimensional transcriptomic data. Starting with ~65,000 mRNA features, we applied unsupervised variance-based filtering and correlation pruning to eliminate low-information genes and reduce redundancy. The applied supervised Select-K Best filtering further refined the feature space. To enhance robustness, we implemented a hybrid selection strategy combining recursive feature elimination (RFE) with random forests and LASSO regression to identify discriminative mRNA features. Finally, these features were then used to construct a gene co-expression similarity network. **Results:** This pipeline reduced approximately 65,000 gene features to a subset of 83 discriminative transcripts, which were then used for network construction to reveal subtype-specific biology. The analysis identified four distinct groups. One group exhibited classical high-grade serous features defined by TP53 mutations and homologous recombination deficiency, while another was enriched for PI3K/AKT and ARID1A-associated signaling consistent with clear cell and endometrioid-like biology. A third group displayed drug resistance-associated transcriptional programs with receptor tyrosine kinase activation, and the fourth demonstrated a hybrid profile bridging serous and endometrioid expression modules. **Conclusions:** This pilot study shows that combining unsupervised and supervised feature selection with network modeling enables robust stratification of ovarian cancer subtypes.

## 1. Introduction

Ovarian cancer is one of the most lethal gynecologic malignancies worldwide, representing the fifth leading cause of cancer-related deaths among women in the United States and the leading cause of death from gynecologic cancers globally [1,2,3]. Each year, more than 19,000 new cases and over 13,000 deaths are reported in the U.S. alone [2], while approximately 314,000 women are diagnosed and 207,000 die from the disease worldwide [3]. The disease is remarkably heterogeneous and is broadly classified into three main histological categories: epithelial tumors, which account for ~90% of all cases; germ cell tumors; and sex cord–stromal tumors [4,5]. Among the epithelial ovarian cancers (EOCs), high-grade serous ovarian carcinoma (HGSOC) is the predominant subtype, representing ~70% of cases, and is distinguished by ubiquitous TP53 mutations and frequent homologous recombination deficiency [6]. Other subtypes include clear cell carcinoma (10%), endometrioid carcinoma (10%), mucinous carcinoma (3–4%), and low-grade serous carcinoma (<5%) [4,5]. These subtypes differ markedly in morphology, genomic alterations, prognosis, and response to therapy [7,8]. Currently, subtype classification relies heavily on histopathology, immunohistochemistry, and targeted sequencing [4,7]. However, these methods are time-intensive, subject to observer variability, and limited in their ability to capture the transcriptional complexity underlying subtype-specific biology, underscoring the need for computational approaches that can leverage genome-wide expression data to refine subtype identification [9,10].

Existing experimental approaches for ovarian cancer subtyping are laborious, often requiring advanced immunohistochemical panels, targeted sequencing, or integrated multi-omic assays [7,8]. Computational methods, in contrast, offer scalable and faster alternatives to resolve subtype identity and extract mechanistic insights from large datasets [11,12]. In particular, transcriptome-wide profiling, and specifically mRNA expression analysis, has become central to ovarian cancer subtype detection [6,10]. mRNA expression patterns differ substantially across subtypes: HGSOC is characterized by transcriptional signatures reflecting DNA repair deficiency and proliferative signaling [6,13]; clear cell carcinomas display enrichment in hypoxia-inducible factor and oxidative stress response pathways [14,15]; endometrioid carcinomas exhibit WNT/β-catenin pathway activation [16]; and mucinous carcinomas display gastrointestinal-like transcriptional programs [14]. These differences make mRNA data a powerful modality to capture the molecular identity of ovarian cancer subtypes and uncover subtle biological groupings that histology alone cannot resolve.

Recent studies have increasingly applied network-based and integrative transcriptomic analyses to decipher molecular heterogeneity in ovarian cancer. For example, Frontiers in Genetics (2022) employed gene co-expression network analysis to identify novel biomarkers and regulatory modules distinguishing tumor from healthy ovarian tissues, emphasizing the potential of network inference for biomarker discovery [17]. Similarly, a 2024 study in the Journal of Ovarian Research used weighted gene co-expression network analysis (WGCNA) to stratify high-grade serous ovarian cancer (HGSOC) and link molecular modules with immune cell infiltration, revealing immunogenomic diversity among subtypes [18]. A large-scale integrative effort published in The Journal of Biological Chemistry (2024) analyzed nearly 2000 ovarian cancer transcriptomes to define consensus molecular subtypes, providing a standardized molecular classification framework [19]. More recently, a 2025 BMC Bioinformatics study introduced a network-based stratification (NBS) approach that integrated somatic mutation and gene expression data to uncover subtype-specific survival associations in multiple cancers including ovarian cancer [20]. Collectively, these studies demonstrate that integrating feature selection, transcriptomic profiling, and network analysis enables the identification of biologically meaningful ovarian cancer subtypes.

Despite its utility, mRNA expression data are inherently high-dimensional, typically encompassing 20,000–65,000 features per sample [7,12,21]. Identifying biologically meaningful subtypes directly from such high-dimensional data is computationally intractable and prone to overfitting [21,22]. Dimensionality reduction methods, such as variance filtering, correlation-based selection, and matrix factorization, are commonly employed to mitigate noise and redundancy [23,24,25,26,27]. However, even after dimensionality reduction, the challenge remains to identify subtype-specific groups that reflect true biological divergence rather than technical artifacts [9,13].

Traditional supervised machine learning approaches, including random forests, support vector machines, and logistic regression, have shown utility in classifying tumors based on established ground truth labels such as benign versus malignant [28,29]. However, these models are not designed to uncover unknown or ambiguous subtypes, as they depend on predefined class labels. As a result, they fail to capture the full spectrum of transcriptional diversity that exists within ovarian cancer subtypes [8,9]. To address this limitation, we propose a multi-stage approach that integrates unsupervised and supervised strategies to reduce both the data complexity and reveal biologically meaningful subgroups.

In this study, we developed a framework that combines unsupervised variance-based feature selection and correlation filtering with supervised methods including wrapper-based approaches and regularization. This hybrid strategy reduces the high-dimensional mRNA feature space to a minimal set of discriminative transcripts, thereby maximizing biological interpretability while preserving subtype-specific signals. Subsequently, we constructed a network model in which each sample is represented as a node and relationships between samples are encoded as edges derived from reduced expression features. This integrated framework enables the discovery of distinct molecular subgroups in ovarian cancer and provides a powerful strategy to improve the fidelity of cell line models and guide subtype-specific research.

## 2. Related Work

The characterization of ovarian cancer subtypes has increasingly relied on computational methods that exploit high-dimensional genomic and transcriptomic data. The Cancer Genome Atlas (TCGA) provided a seminal framework by integrating multiple data modalities and identifying four molecular subtypes of high-grade serous ovarian carcinoma (HGSOC) using unsupervised clustering of mRNA expression profiles [6]. Similarly, Tothill et al. applied large-scale gene expression profiling to serous and endometrioid ovarian cancers and revealed distinct groups associated with prognosis [10]. These early studies underscored the potential of transcriptome-wide data to resolve molecular heterogeneity but also highlighted the challenges of analyzing such high-dimensional information.

Network-based methods have emerged as powerful tools for uncovering subtype-specific biology by considering gene–gene or sample–sample relationships. Zhang et al. applied network-based survival analysis, using co-expression networks to identify ovarian cancer subtypes associated with patient outcomes [30]. Hollis et al. demonstrated that molecular stratification of endometrioid ovarian carcinoma could be achieved by combining transcriptomic data with gene interaction networks, revealing biologically coherent subgroups predictive of survival [31]. Other studies have leveraged pathway-informed networks, where nodes represent genes and edges represent functional relationships, to refine ovarian cancer subtype classification and identify driver modules [32,33]. These approaches highlight the value of incorporating network structure into classification tasks, as they provide biological interpretability beyond clustering alone.

Machine learning has also been widely applied to subtype discovery in ovarian cancer. Verhaak et al. identified prognostic gene signatures in HGSOC using supervised learning methods, linking transcriptional patterns to patient survival [34]. Domcke et al. benchmarked ovarian cancer cell lines against primary tumors using integrative genomic comparisons, underscoring the importance of appropriate training data when applying supervised classifiers [35]. More broadly, Libbrecht and Noble reviewed the use of machine learning in genomics, emphasizing that while supervised models are effective for binary classification tasks, they are not inherently suited for discovering novel subtypes [36]. Kourou et al. further demonstrated that machine learning approaches can improve cancer prognosis and prediction but cautioned that high-dimensionality and class imbalance remain major barriers [37].

Hybrid approaches that combine dimensionality reduction with network-based learning have proven particularly effective. Lee and Seung introduced non-negative matrix factorization (NMF) as a method to decompose high-dimensional expression data into biologically meaningful features [24]. Brunet et al. extended NMF for cancer subtype discovery, demonstrating its capacity to uncover coherent molecular patterns [27]. More recent advances incorporate correlation filtering, feature selection, and network inference to refine feature spaces and construct sample similarity networks, thereby enabling the robust stratification of tumors into biologically relevant groups [12,23].

Despite these advances, existing methodologies often suffer from two key limitations. First, purely unsupervised approaches can identify clusters but may fail to highlight the most discriminative features driving subtype divergence. Second, traditional supervised methods require predefined labels, which restrict their utility for discovering new or ambiguous subtypes. Our proposed methodology addresses these limitations by combining unsupervised variance-based feature selection with supervised statistical and wrapper methods to reduce the high-dimensional mRNA feature space into a minimal and biologically informative set. By integrating network modeling, we represent each sample as a node and their relationships as edges, enabling subtype identification that is both computationally tractable and biologically interpretable. This multi-stage framework advances prior work by balancing feature selection, interpretability, and network-level context, thereby offering a more robust strategy for uncovering ovarian cancer subtypes.

## 3. Materials and Methods

**Figure 1:**
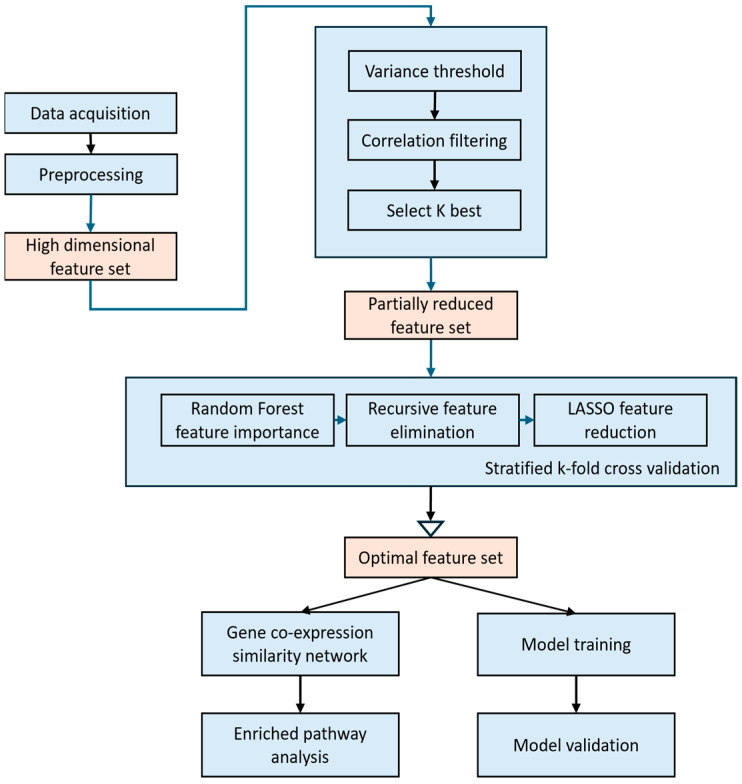
Workflow of the multi-stage feature selection and analysis framework. The pipeline begins with data acquisition, preprocessing, and the generation of a high-dimensional mRNA feature set. Initial unsupervised reduction is performed using variance thresholding, correlation filtering, and K-best selection to derive a partially reduced feature set. This is followed by a hybrid supervised feature selection stage integrating random forest feature importance, recursive feature elimination (RFE), and LASSO regression with stratified k-fold cross-validation, resulting in an optimal feature set. The selected features are then used for downstream analyses, including gene co-expression similarity network construction, enriched pathway analysis, model training, and model validation, enabling robust subtype characterization and biological interpretation of ovarian cancer.

To address the challenge of analyzing high-dimensional transcriptomic data, we developed a multi-stage feature selection and network-based analysis framework (as shown in Figure 1). The methodology begins with data acquisition, where ovarian cancer cell lines were profiled for transcriptomic alterations. This was followed by preprocessing, including RNA extraction, quality control, and sequencing, to generate high-dimensional mRNA expression data. Then, we identified the most informative mRNA features and minimized redundancy, using unsupervised and supervised strategies. The optimized feature set was subsequently applied to model training, validation, and biological interpretation through co-expression networks and pathway enrichment analysis.

### 3.1. Experimental Design

Human ovarian cancer cell lines SKOV3, TOV21G and OVCAR-3 were obtained from ATCC (Manassas, VA, USA). Additional ovarian cancer cells IGROV-1, COV364.2 were purchased from Sigma-Aldrich (St. Louis, MO, USA). The OVCAR-5 cell line was obtained from Cell BioLabs (San Diego, CA, USA), and OVCAR-8 cells were purchased from Creative Biolabs (Shirley, NY, USA). The immortalized human normal ovarian epithelial cell line, HOSE-T80, was generously provided by Dr. John Davis (University of Nebraska medical Center, Omaha, NE, USA). Immortalized human normal ovarian epithelial cells HIO-80 were kindly provided as a gift from Dr. Andrew K. Godwin (University of Kansas Medical Center, Kansas City, KS, USA). Human normal ovarian epithelial cells (HOSE-T80 and HIO-80) were cultured in a 1:1 mixture of Medium 199 and MCDB 105. All human ovarian cancer cell lines (SKOV3, TOV21G, OVACR-3, IGROV-1, COV362.4, OVCAR-5, and OVCAR-8) were maintained in DMEM. All media were supplemented with 10% fetal calf serum and penicillin/streptomycin (100 ug/mL), and cells were incubated at 37 °C in a humid atmosphere of 5% CO_2_.

### 3.2. Total RNA Extraction and RNA Sequencing

Total RNA was extracted from ovarian cancer and noncancer cell lines using the GeneJET™ RNA Purification Kit (cat. # K0731; Thermo Fisher Scientific, Waltham, MA, USA), following the manufacturer’s recommended protocol. Each line had three technical replicates for a total of seven ovarian cancer RNA samples. Two noncancer ovarian epithelial control cell lines were also processed in triplicate for this analysis. Additionally, eight publicly available samples from NCBI’s SRA [38], representing healthy control cell lines, were analyzed to increase the control sample number. Of the eight SRA samples, four were fallopian tube secretory epithelial cells (FT33tag, FT194tag, FT33shp, FT237shp), and four were ovarian surface epithelial cells (IOSE364, IOSE386, IOSE80, IOSE primary cells). In total, 27 mRNA-seq libraries were sequenced on the Illumina NovaSeq platform (150bp paired-end reads) to an average read depth of 50.17 million reads per sample (Illumina Inc., San Diego, CA, USA). Raw fastq reads underwent adapter trimming and low-quality bases were removed using the BBMap suite’s BBDuk function [39]. Trimmed reads were then aligned to GENCODE human genome transcripts (release 47 GRCh38.p14) using STAR aligner [40]. Transcript quantification was conducted using Salmon [41]. Transcript quantities from Salmon were used for all machine learning methodologies described below.

### 3.3. Feature Selection

Step 1: Variance threshold filtering: Variance-based filtering is a commonly used unsupervised method for removing uninformative features that exhibit minimal variation across samples. Low-variance genes are unlikely to contribute meaningfully to clustering or classification tasks because they provide little discriminatory power between groups [23,42]. In this study, we applied a variance threshold of 10, which retained only genes with substantial expression variability across samples. This reduced the original 63,000 mRNA features to 21,000 features approximately, thereby decreasing dimensionality while preserving biologically variable signals.

Step 2: Correlation filtering: Gene expression datasets often contain redundant features due to high correlation between genes. Such redundancy can introduce noise, inflate computational complexity, and hinder the identification of independent discriminative features [26,43]. To address this, we performed pairwise correlation analysis across the 21,000 genes. For each pair of genes with a Pearson correlation coefficient greater than 0.90, one gene was removed. This correlation-based filtering step reduced the dataset to 8000 features approximately, ensuring a more efficient and less redundant feature space for downstream modeling.

Step 3: Supervised feature selection (SelectKBest): Although our overall framework was designed for subtype discovery, an intermediate supervised feature selection step was employed to prioritize features most likely to exhibit discriminative value. We implemented the SelectKBest method, which evaluates individual features using statistical tests of association with phenotype labels such as chi-squared or ANOVA F-statistics [44]. This supervised filter enabled the ranking of features by relevance and reduced the dataset to 5000 candidate mRNA features. By narrowing the feature space to genes with significant associations, this step improved the balance between dimensionality reduction and the retention of biological signals.

Step 4: Hybrid feature selection:

Step 4.1: Random forest feature importance (RF feature importance): Random forest (RF) was applied as an embedded feature selection method that leverages an ensemble of decision trees to evaluate variable importance. By computing the mean decrease in Gini impurity or permutation importance, random forest identifies features contributing most strongly to classification accuracy. This method is advantageous in high-dimensional transcriptomic data because it captures nonlinear interactions and is relatively robust to noise [44]. Applying random forest reduced the initial 5000 mRNA features to the top 2000 most informative transcripts, effectively eliminating redundant or low-importance variables while retaining biologically significant signals.

Step 4.2: Recursive feature elimination (RFE)**:** Recursive feature elimination (RFE) is a wrapper-based method that iteratively builds a model, ranks features by importance, and removes the least important features until an optimal subset is obtained [45]. We implemented RFE with support vector machines to refine the 2000 RF-selected features. Through iterative elimination, the feature space was further reduced to 1000 transcripts, prioritizing genes with the greatest discriminative power. RFE is particularly well-suited for genomic datasets, as it integrates feature importance directly into the training process, enabling biologically informative features to be retained.

Step 4.3: LASSO regularization: To achieve the final selection, we applied Least Absolute Shrinkage and Selection Operator (LASSO) regression. LASSO introduces an L1 penalty that shrinks coefficients of irrelevant variables to zero, effectively performing embedded feature selection [46]. Applied to the 1000 RFE-selected transcripts, LASSO reduced the set to the top 500 mRNAs, representing the most discriminative features. This approach has demonstrated particular utility in genomics, where the number of features far exceeds the number of samples, and has been successfully applied in genome-wide association studies and transcriptomic modeling [47].

Step 5: Final feature set: By integrating random forest feature importance, RFE, and LASSO within a hybrid feature selection framework, we aimed to identify a consensus set of discriminative mRNA features. To ensure robustness, feature selection was performed within a stratified k-fold cross-validation setting, and only those mRNAs that consistently appeared across all folds were retained. This strategy minimized overfitting and emphasized reproducible signals, yielding a reduced feature set that represents the most biologically informative transcripts for downstream grouping and network modeling

### 3.4. Gene Co-Expression Similarity Network

Representing transcriptomic data as a gene co-expression similarity network offers significant advantages in uncovering functional relationships and coordinated biological processes. In such networks, nodes correspond to individual genes, and edges represent statistically significant correlations between their expression profiles across samples. This approach provides an intuitive and biologically interpretable representation of the transcriptome, highlighting connections that are often obscured in raw high-dimensional data. The construction of a co-expression similarity network relies on two key properties: the strength of pairwise expression correlations between genes and the emergent topological organization of the resulting network. Initially, the network is assembled by calculating pairwise Pearson correlations among the selected transcripts, with edges drawn between genes exceeding a defined correlation threshold. This strategy ensures that only strongly co-expressed genes are retained, thereby emphasizing functionally relevant associations.

The steps involved in this process are illustrated below.

Preliminaries:

The methodology for creating the correlation network involves several assumptions:

Postulation 1: N represents the total number of subjects included in the study.

Postulation 2: K denotes the number of proposed features utilized to construct the correlation network.

Postulation 3: Pi and Pj represent randomly selected individual subjects from the population of N subjects. The indices (i,j) satisfy the conditions (i,j) ≤ N and (i,j) > 0, ensuring valid subject indices within the population.

Postulation 4:. CM refers to the correlation matrix generated by computing ρ[i,j] for all valid subject pairs (i,j).

Postulation 5: T represents a predefined threshold set by the user to determine the minimum strength of correlation required for constructing the complex network.

Postulation 6: SM denotes the significance matrix obtained after applying the threshold T to the correlation coefficients in CM.

Procedure:

To create a similarity network representing the relationships between subjects based on their expression patterns, the following steps are undertaken:

Compute pairwise Pearson correlation:

- Calculate the Pearson pairwise correlation coefficient ρ between each pair of subjects Pi and Pj, where i and j range from 1 to N.
- ρ[i,j] represents the correlation coefficient between subjects Pi and Pj.

Generate correlation matrix (CM):

- Create a correlation matrix (CM) of size N × N, where N is the number of subjects in the study.
- Each element CM[i,j] of the matrix represents the ρ value between subjects Pi and Pj.

Set threshold (T) for correlation strength:

- Choose a predefined threshold value T to determine the strength of the correlation required for constructing the similarity network.
- A threshold above 0.5 is considered strong, but the specific value can be adjusted based on the desired criteria [48,49]. Typically, a correlation threshold of ≥0.5 can be applied to define significant connections between genes or samples. This value was chosen based on its common usage in gene co-expression network studies, where moderate correlation cutoffs effectively balance network sparsity and biological interpretability. Prior work has demonstrated that such thresholds capture stable co-expression patterns reflective of shared regulatory or functional relationships while minimizing false-positive edges arising from random noise [50,51]. The selection of an appropriate correlation threshold is critical in co-expression network construction. Best practices recommend choosing a threshold that maintains sufficient network connectivity while minimizing spurious associations, often determined empirically through sensitivity testing or guided by prior studies reporting biologically stable network structures.

Generate significance matrix (SM):

- Create a significance matrix (SM) based on the correlation values in CM using the formula:

$$SM[i,j]=\{\begin{array}{cc} 1, & if\;CM[i,j]\geq T\\ 0, & Otherwise \end{array}$$

- SM is an adjacency matrix, where each element SM[i,j] determines whether an edge should be present between subjects Pi and Pj in the final network.

Generate similarity network:

- Based on the values in the SM matrix, it generates the similarity network.
- For each pair of subjects (Pi,Pj) where SM[i,j] > T (indicating a significant correlation), create an edge connecting Pi and Pj in the network.

### 3.5. Illustrative Example

To illustrate the process of constructing a complex network from extracted feature information, consider a dataset with six subjects, denoted as X1, X2, …, X6. Four features, F1, F2, F3, and F4, are extracted from raw sensor data collected from these subjects. The first step in the process is to calculate the Pearson pairwise correlation coefficients, resulting in a correlation matrix (CM), which is a table where each entry represents the correlation coefficient between a pair of subjects. Consider the example correlation matrix (CM) shown in Table 1. For instance, the entry in the first row and second column of the CM represents the correlation coefficient between X1 and X2. Similarly, the entry in the third row and fourth column represents the correlation coefficient between X3 and X4.

The next step is to choose a threshold T. Assume we choose a threshold T = 0.7. In general, choosing a threshold T to generate a network from a correlation matrix containing Pearson correlation coefficients is a critical step that influences the structure and interpretation of the resulting network. First, the threshold should be selected based on the strength of the relationships you want to capture; typically, higher thresholds are used to focus on stronger correlations, ensuring that only meaningful connections are represented. Second, the distribution of correlation values across the dataset should be considered; a threshold should be chosen so that it balances the inclusion of relevant correlations while avoiding the creation of an overly dense or sparse network. Finally, the threshold may also be influenced by domain-specific considerations or prior knowledge, where certain correlation strengths are known to be significant in the context of the study, guiding the selection of an appropriate cutoff value. Using this threshold, we generated a significance matrix (SM), which is an adjacency matrix where entries are set to 1 if the corresponding correlation coefficient is greater than or equal to T, and 0 otherwise. By applying the chosen threshold T, the resultant SM is shown in Table 2.

**Figure 2:**
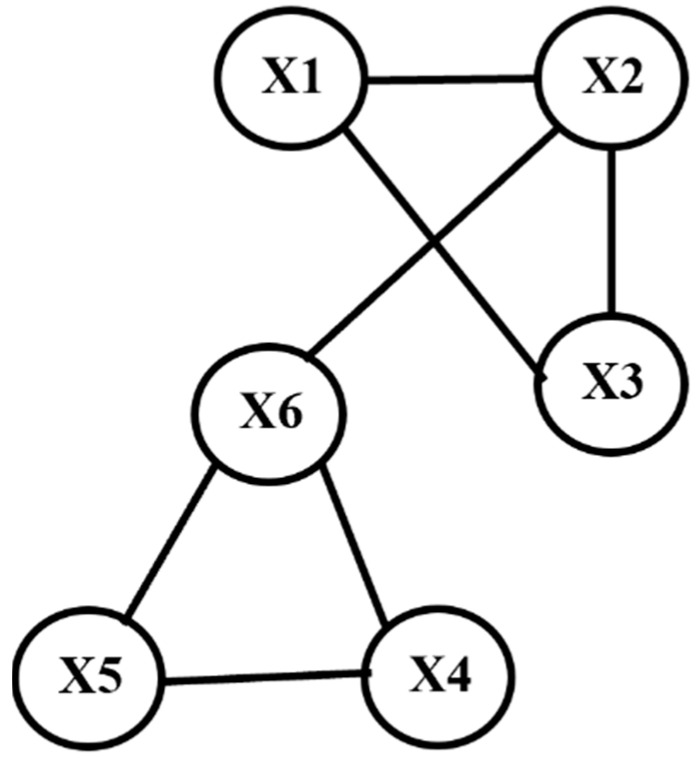
Network representation derived from the significance matrix. This figure depicts a network graph constructed from the significance matrix, which is equivalent to an adjacency matrix. Nodes represent features (X1–X6), and edges indicate significant correlations between features after applying a threshold of T = 0.7. For example, since the correlation between X1 and X2 exceeded the threshold, the corresponding entry was marked as 1 in the significance matrix, resulting in an edge between X1 and X2 in the network.

The significance matrix (SM) is inherently an adjacency matrix. We converted this matrix into a similarity network representation, where nodes represent subjects, and edges represent significant correlations between subjects (with weights greater than the threshold). The similarity network representation for Table 2 is shown in Figure 2. This example demonstrates the process of converting feature extraction data into a similarity network representation, thereby visualizing the underlying hidden intrinsic relationships.

## 4. Results

### 4.1. Identification of Discriminative mRNAs Using Feature Selection

**Figure 3:**
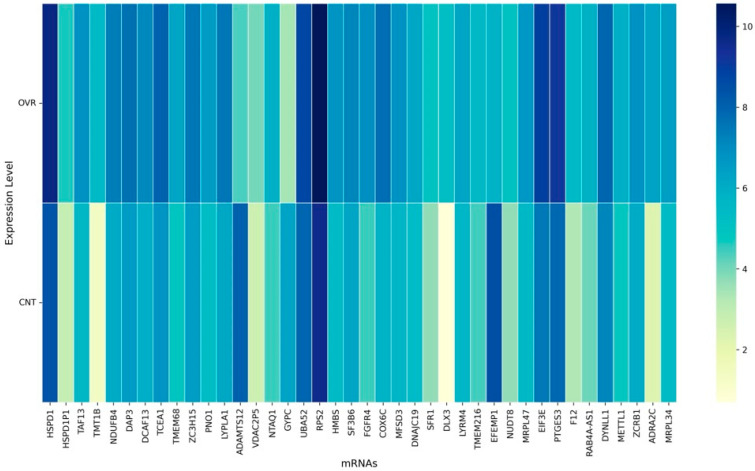
Differential expression of selected mRNAs between the ovarian cancer (OVR) and control (CNT) samples—Panel 1. The heatmap illustrates log-transformed expression levels of a subset of discriminative mRNAs across the ovarian cancer (OVR) and control (CNT) groups. Each column represents an individual mRNA, and rows correspond to sample categories. Distinct patterns of upregulation (darker shades) and downregulation (lighter shades) are evident, reflecting subtype-specific transcriptional changes associated with ovarian cancer.

**Figure 4:**
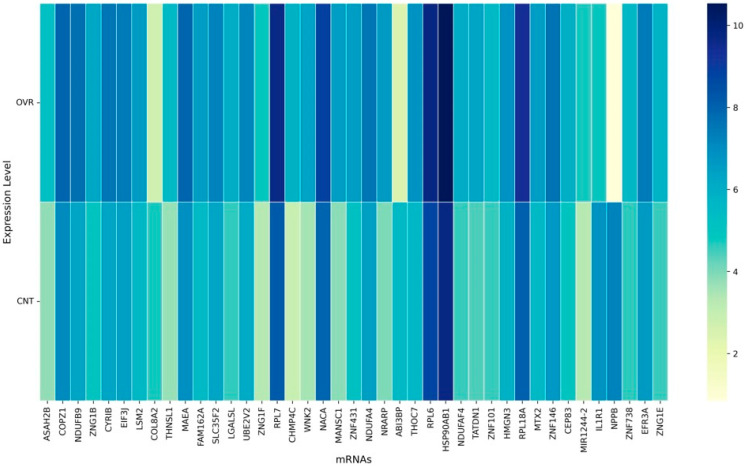
Differential expression of selected mRNAs between the ovarian cancer (OVR) and control (CNT) samples—Panel 2. This heatmap displays a complementary set of mRNAs identified through feature selection, highlighting their expression variation across the ovarian cancer (OVR) and control (CNT) groups. Similar to Panel 1, the grouping of expression intensities revealed consistent separation between the cancerous and control states, reinforcing the discriminatory potential of the selected mRNA features.

Application of the multi-stage feature selection framework reduced the initial transcriptome of ~63,000 mRNAs to a final panel of 83 discriminative features (as shown in Figure 3 and Figure 4). This condensed panel captured the most biologically informative variation across ovarian cancer samples, providing a foundation for classification and biological interpretation.

The final 83-gene panel included multiple transcripts involved in mitochondrial energy metabolism and oxidative phosphorylation, a hallmark of ovarian cancer metabolic reprogramming. Several mitochondrial genes were strongly overexpressed in ovarian cancer relative to the controls, including NDUFB4, NDUFB9, NDUFA4, DNAJC19, COX6C, MRPL34, MRPL47, and NDUFAF4, highlighting enhanced mitochondrial respiratory chain activity [52,53]. In contrast, ABI3BP was markedly under expressed, consistent with prior reports suggesting a tumor-suppressive role.

Genes related to ribosomal structure and translation were also enriched and consistently overexpressed. These included RPL6, RPL7, RPL18A, RPS2, EIF3E, EIF3J, and UBA52, reflecting the elevated protein synthesis demands of cancer cells [54,55]. Similarly, regulators of RNA processing and splicing such as TCEA1, SF3B6, ZCRB1, PNO1, and DCAF13 were overexpressed, indicating dysregulation of mRNA metabolism and splicing fidelity in ovarian cancer [56,57].

Several genes involved in oncogenic signaling and extracellular matrix (ECM) remodeling were also identified. FGFR4, NRARP, and IL1R1 were included, with FGFR4 and NRARP overexpressed, while IL1R1 was under expressed. Notably, FN1 and EFEMP1, two critical ECM regulators, were under expressed in ovarian cancer relative to the controls, supporting their role in impaired adhesion and aggressive tumor phenotypes. ADAMTS12 also showed downregulation, consistent with its role in ECM turnover. Interestingly, the panel also contained stress response and chaperone-associated genes. HSPD1, HSPD1P1, and HSP90AB1 were strongly overexpressed, reflecting enhanced protein folding and stress adaptation. Likewise, DAP3 and DYNLL1 (apoptosis and cytoskeletal regulators) were overexpressed, underscoring the survival and migration advantages conferred to tumor cells [58,59,60,61,62,63,64].

The overall expression patterns emphasize two distinct groups: (1) a large set of overexpressed genes including mitochondrial, ribosomal, and chaperone-associated transcripts that support metabolic rewiring, rapid growth, and stress resistance; and (2) a smaller set of under expressed genes, such as FN1, EFEMP1, ADAMTS12, ABI3BP, and IL1R1, which represent loss of adhesion and tumor suppressor-like functions. These findings suggest that the hybrid feature selection pipeline not only identified computationally stable biomarkers, but also converged on biologically meaningful signatures of ovarian cancer [65,66,67].

### 4.2. Model Training and Validation Performance

To rigorously assess the predictive performance of the classification models, we employed stratified k-fold cross-validation, which preserves the proportion of class labels in both the training and validation subsets. This approach minimizes sampling bias and ensures that each fold provides a representative balance of ovarian cancer and control samples. Due to the limited sample size inherent to this pilot study, the same dataset was used for both the training and testing phases while maintaining separation between folds to prevent data leakage. The dataset was partitioned into 80% for training and 20% for testing within each iteration of the cross-validation procedure. To mitigate class imbalance, we applied the synthetic minority oversampling technique (SMOTE) exclusively on the training data [68]. SMOTE synthetically generates minority class instances by interpolating between existing samples, thereby equalizing class distributions and enhancing model generalization. This strategy ensured that the performance evaluation remained statistically sound despite the small dataset size and the exploratory nature of this study. Furthermore, each sample, including replicates, was taken as an individual sample instead of averaging the triplicate.

During the training phase, both logistic regression and random forest models achieved perfect classification performance across all evaluation metrics (as shown in Table 3), with accuracy, sensitivity, specificity, F1 score, and AUC consistently at 1.00 ± 0.00 (95% CI: 1.00–1.00). AdaBoost also demonstrated excellent performance with an accuracy of 0.97 ± 0.06, sensitivity of 1.00, specificity of 0.90, F1 score of 0.98 ± 0.04, and AUC of 0.95 ± 0.11, reflecting robust discriminatory power with minimal variance. Decision tree models yielded an accuracy of 0.91 ± 0.13, sensitivity of 0.92 ± 0.18, specificity of 0.93 ± 0.15, F1 score of 0.93 ± 0.11, and AUC of 0.93 ± 0.10, indicating reliable but slightly more variable classification performance compared with ensemble methods. XGBoost achieved an accuracy of 0.86 ± 0.20, with a sensitivity of 0.81 ± 0.32, specificity of 0.90 ± 0.22, F1 score of 0.84 ± 0.26, and AUC of 0.86 ± 0.20, highlighting its capacity to achieve good performance but with larger variability and wider confidence intervals. The SVM classifier demonstrated high accuracy at 0.91 ± 0.13, perfect sensitivity (1.00), specificity of 0.77 ± 0.32, F1 score of 0.94 ± 0.09, and AUC of 0.98 ± 0.04. While sensitivity remained strong, specificity fluctuated considerably, suggesting class imbalance sensitivity during training.

On validation, the logistic regression and random forest models again maintained perfect performance across all metrics (as shown in Table 4), reaffirming their robustness and stability. AdaBoost preserved high classification ability with an accuracy of 0.97 ± 0.06, sensitivity of 1.00, specificity of 0.90, F1 score of 0.98 ± 0.04, and AUC of 0.95 ± 0.11, consistent with the training results. Decision tree validation performance improved compared with training, with an accuracy of 0.97 ± 0.06, sensitivity of 1.00, specificity of 0.93 ± 0.15, F1 score of 0.98 ± 0.05, and AUC of 0.97 ± 0.07, reflecting its strong generalization capability when applied to unseen data. XGBoost also improved during validation, yielding an accuracy of 0.91 ± 0.13, sensitivity of 0.86 ± 0.22, specificity of 1.00, F1 score of 0.91 ± 0.14, and AUC of 0.93 ± 0.11. These results suggest that XGBoost, despite variability in training, demonstrated greater robustness on validation. In contrast, SVM exhibited reduced performance during validation, with an accuracy of 0.83 ± 0.12, specificity of 0.60 ± 0.28, and F1 score of 0.88 ± 0.08, although sensitivity remained perfect (1.00) and AUC retained maximum discriminatory capacity (1.00). Overall, logistic regression and random Forest consistently provided perfect classification in both training and validation phases.

### 4.3. Similarity Network Representation

**Figure 5:**
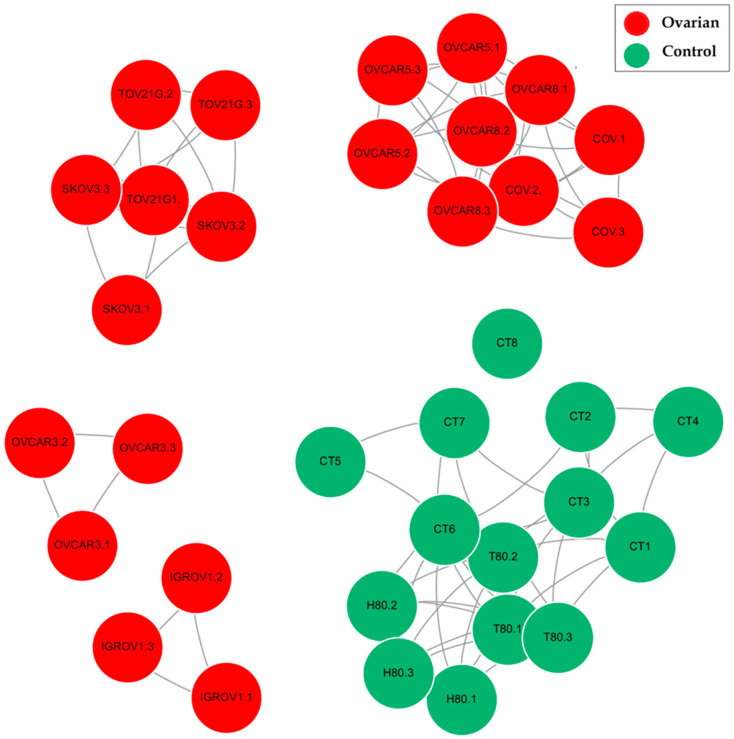
Gene co-expression similarity network constructed from selected mRNA features. In this network, nodes represent individual genes and edges denote significant pairwise correlations above the defined threshold, reflecting strong co-expression relationships. Nodes colored in red represent ovarian cancer cell lines, whereas nodes colored in green correspond to control cell lines. The figure demonstrates a clear separation between the ovarian and control groups, with distinct subgroups observable within the ovarian cancer group, suggesting potential subtype-specific expression patterns.

**Figure 6:**
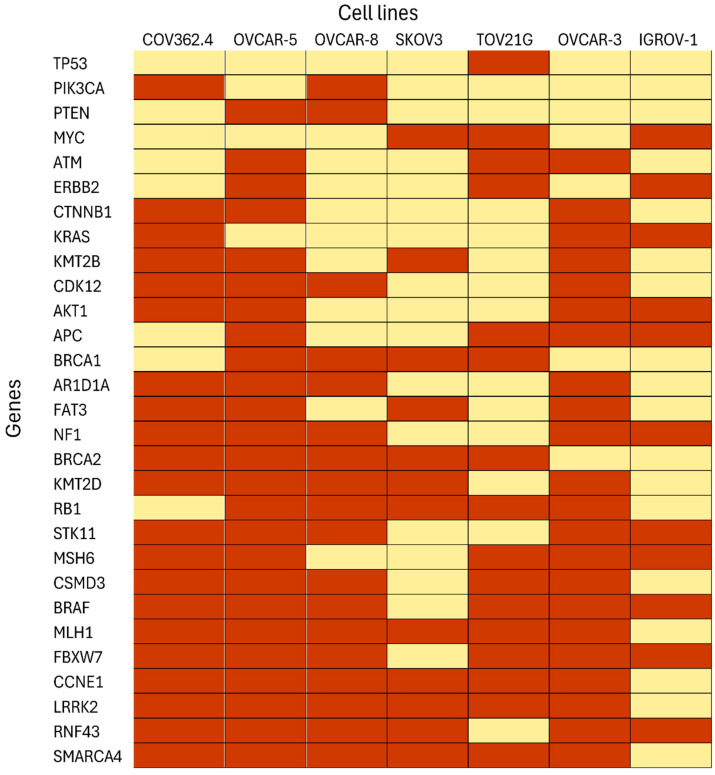
Mutations across ovarian cancer cell lines. The figure summarizes the mutation landscape of the seven ovarian cancer cell lines represented in this study. Rows correspond to genes, and columns represent cell lines, ordered into four biologically defined groups: Group 1 (COV362.4, OVCAR-5, OVCAR-8), Group 2 (SKOV3, TOV21G), Group 3 (OVCAR-3), and Group 4 (IGROV-1). Each filled cell (yellow) indicates the presence of a mutation in the corresponding gene, while empty cells (red) represent wild-type status. Genes are ordered by overall mutation frequency across cell lines to highlight recurrently altered pathways. This visualization underscores the predominance of TP53 mutations across most models, alterations in BRCA1/2 and DNA damage repair genes in Group 1 and Group 3, enrichment of ARID1A, PIK3CA, and PTEN in Group 2, and distinct MYC and SMARCA4 alterations in OVCAR-3 and IGROV-1, respectively.

Unsupervised feature selection combined with network modeling of the mutational data stratified the seven ovarian cancer cell lines into four biologically coherent groups (as shown in Figure 5). Group 1 comprised COV362.4, OVCAR-5, and OVCAR-8. Group 2 consisted of SKOV3 and TOV21G. Group 3 was represented solely by OVCAR-3, while IGROV-1 was assigned to Group 4. The resulting classification recapitulates known histopathological and molecular features of these cell lines and highlights the biological heterogeneity within models commonly used for ovarian cancer research. In addition, we incorporated genetic alteration (mutation/deletion/amplification) (shown in Figure 6) across all cancer lines and examined both commonalities and subtype-specific variations between the groups. Notably, the mutational patterns identified through this analysis were consistent with previously reported findings in the literature, providing further validation of the biological relevance of our grouping strategy.

Group 1 was characterized by canonical features of high-grade serous ovarian carcinoma (HGSOC). All three cell lines carried TP53 mutations, which represent the defining molecular hallmark of this subtype [6]. COV362.4 was BRCA1-deficient, consistent with homologous recombination deficiency, while OVCAR-5 and OVCAR-8 exhibited additional alterations in MYC amplification and PI3K pathway genes including PIK3CA and PTEN. Network modeling positioned these lines within the serous core module enriched for DNA damage response deficiency and chromosomal instability, consistent with prior genomic characterizations of HGSOC cell lines [12,35].

In contrast, Group 2, consisting of SKOV3 and TOV21G, clustered apart from the HGSOC core. TOV21G carried mutations in ARID1A and PIK3CA, which are established drivers of ovarian clear cell carcinoma [7]. SKOV3, although historically classified as serous, lacked TP53 mutations and instead exhibited alterations in PIK3CA, PTEN, AKT1, and ARID1A. This mutational spectrum aligns SKOV3 more closely with endometrioid or clear cell-like biology than with HGSOC [1,69]. Feature selection emphasized PI3K/AKT and ARID1A-driven oncogenic signaling as the unifying signatures of this group, and network modeling confirmed their placement within a distinct non-HGSOC module.

Group 3, which included only OVCAR-3, was separated from the other HGSOC cell lines despite sharing a TP53 mutation. OVCAR-3 harbored a unique constellation of alterations including BRCA2 and CDK12 mutations, MYC amplification, and ERBB2 pathway involvement. These features reflect the clinical history of OVCAR-3, which was derived from a heavily pretreated, chemoresistant patient [70]. Network analysis placed OVCAR-3 in an outlier serous module enriched for multidrug resistance and receptor tyrosine kinase signaling, consistent with its distinct phenotype as a model of therapy-refractory disease.

Group 4 contained IGROV-1, which grouped separately due to its hybrid mutation profile. IGROV-1 displayed alterations in TP53, BRCA1/2, and ATM, which are characteristic of serous carcinomas, but also harbored mutations in CTNNB1, APC, KMT2D, and RB1, more typically observed in endometrioid ovarian cancers. This duality reflects its origin from an endometrioid carcinoma, later reclassified based on serous-like genomic features [71]. In the network model, IGROV-1 showed connections to both DNA repair-deficient serous modules and WNT/β-catenin-driven endometrioid modules, justifying its isolation into a separate group.

Taken together, the integration of feature selection and network modeling demonstrated that COV362.4, OVCAR-5, and OVCAR-8 form a cohesive HGSOC core group defined by TP53 mutations and homologous recombination deficiency. SKOV3 and TOV21G constitute a non-HGSOC group unified by PI3K/ARID1A-driven signaling. OVCAR-3 represents a distinct drug-resistant HGSOC outlier characterized by multidrug resistance and receptor tyrosine kinase pathway activity, while IGROV-1 reflects hybrid biology bridging serous and endometrioid lineages. This classification underscores the biological heterogeneity of ovarian cancer cell lines and provides a framework for selecting appropriate preclinical models for subtype-specific studies.

To further strengthen the robustness of our findings, a subset of the selected genes—TMT1B, COX6C, ADAMTS12, and ABI3BP—were subjected to biological validation using droplet digital PCR (ddPCR) technology [72]. This highly sensitive and quantitative method confirmed the differential expression patterns observed in the transcriptomic analysis, thereby supporting the biological relevance of these candidates in ovarian cancer.

## 5. Discussion, Limitations, and Future Directions

In this pilot study, we applied a multi-stage feature selection and network analysis framework to high-dimensional transcriptomic data to stratify ovarian cancer cell lines into biologically meaningful subtypes. Starting with over 63,000 mRNA features, our pipeline reduced dimensionality through variance filtering, correlation pruning, supervised selection, and hybrid methods combining recursive feature elimination (RFE) and LASSO regression. The final 83 transcripts spanned diverse cellular processes, including mitochondrial metabolism, ribosome biogenesis, RNA processing, extracellular matrix remodeling, and oncogenic signaling, hallmarks of ovarian cancer such as metabolic reprogramming, aberrant protein synthesis, and dysregulated signaling. Several selected mRNAs, including FN1, EFEMP1, and FGFR4, have been previously linked to ovarian cancer, validating the biological relevance of the reduced panel [65,73,74].

The stratification of ovarian cancer cell lines into four groups highlights concordance with both the established classifications and discrepancies that warrant careful interpretation. Group 1, which includes COV362.4, OVCAR-5, and OVCAR-8, was grouped together under a high-grade serous ovarian cancer (HGSOC)-like profile by the similarity network. However, a key limitation emerged with the classification of OVCAR-8. While the network-based grouping suggested alignment with HGSOC, prior literature indicates that OVCAR-8 more closely resembles a low-grade serous ovarian cancer (LGSOC) model due to the presence of KRAS and ERBB2 mutations, genetic alterations that are more typical of low-grade tumors [75,76]. Group 2, consisting of SKOV3 and TOV21G, represents clear cell ovarian cancer (CCOC), which is characterized clinically by poor chemotherapy response rates (~15%) and resistance to platinum-based therapies. Group 3, represented by OVCAR-3, remains consistent with an HGSOC subtype, but its strong androgen and estrogen receptor expression highlights a distinctive hormonal regulatory profile. Group 4, IGROV-1, exhibits mixed histology, predominantly endometrioid with serous clear cell components and undifferentiated regions. Unlike OVCAR-3, IGROV-1 is hormone receptor-negative and is defined by a hypermutated phenotype, aligning with prior observations of genomic instability in this line [71,77].

In addition to transcriptomic features, the mutational landscape of the seven ovarian cancer cell lines (shown in Figure 6) further underscores the biological heterogeneity captured in our stratification. As expected, TP53 alterations were nearly universal across all models, consistent with its well-established role as a hallmark driver of high-grade serous ovarian carcinoma. Mutations in DNA damage repair genes such as BRCA1, BRCA2, and ATM were distributed unevenly, with IGROV-1, COV362.4, and OVCAR-3 harboring such defects, thereby reinforcing the association between homologous recombination deficiency and serous-like phenotypes. In contrast, SKOV3 and TOV21G exhibited frequent alterations in ARID1A, PIK3CA, and PTEN, aligning with canonical pathways implicated in clear cell and endometrioid tumors. Notably, oncogenic signaling through the KRAS and ERBB2 axes was enriched in SKOV3, TOV21G, and OVCAR-5, suggesting divergent drivers of proliferation within these subtypes. OVCAR-3 stood out for its unique enrichment in MYC amplification alongside extracellular matrix-associated transcriptional programs, reinforcing its distinct molecular identity and reported chemoresistant behavior. IGROV-1 harbored rare mutations such as SMARCA4 and LRRK2, further supporting its hybrid classification between serous and endometrioid lineages. Taken together, the mutational information complements our transcriptomic findings by demonstrating that the subtype-specific groupings derived from gene expression are underpinned by coherent patterns of pathway-level alterations, many of which have been previously validated in large-scale genomic studies such as TCGA.

This study has several implications. First, it highlights the potential of systematic feature reduction and network modeling to resolve transcriptional heterogeneity and provide interpretable biomarkers for ovarian cancer subtype classification. Second, it emphasizes that even in a pilot-scale study with a limited number of cell lines, biologically coherent patterns can be uncovered that correspond to established histotypes. Finally, it provides a proof-of-concept framework that can be extended to larger patient-derived datasets, where subtype discovery and classification remain critical for guiding precision therapy. A comprehensive list of enriched pathways, gene sets, and statistical outputs is provided in the Supplementary Materials for further reference.

Nevertheless, this work is not without limitations. The use of a small panel of cell lines limits generalizability, and cell lines may not fully capture the complexity and tumor microenvironment interactions present in patient tumors. An additional constraint is that only two control cell lines were available at the time of the experiments, preventing us from including an equal number of control samples. To mitigate this imbalance, we supplemented the analysis with control data obtained from the TCGA public dataset. Future work will focus on expanding this framework to large-scale, patient-derived transcriptomic datasets to validate the subtype-specific molecular signatures identified in this study. Integrating genomic, proteomic, and clinical data from public repositories and prospective patient cohorts will allow for the assessment of the reproducibility and prognostic relevance of these subtypes. While our feature selection approach was designed to minimize overfitting, the perfect classification performance observed in some models may reflect the limited sample size rather than universal generalizability. Validation in larger, independent cohorts, including patient-derived xenografts and clinical tumor samples, will be essential to confirm the robustness and clinical relevance of the selected gene panel. Furthermore, while our framework identified subtype-specific gene sets, functional validation will be necessary to determine the causal role of these transcripts in ovarian cancer progression and therapeutic response.

## 6. Conclusions

In this pilot study, we applied a multi-stage feature selection and network modeling framework to high-dimensional mRNA expression data from ovarian cancer cell lines. Beginning with ~63,000 transcripts, systematic filtering and refinement through random forest feature importance, recursive feature elimination, and LASSO regression identified a compact panel of 83 discriminative mRNAs. These genes captured key biological processes including mitochondrial metabolism, ribosome biogenesis, RNA processing, extracellular matrix remodeling, and oncogenic signaling pathways. Integration of mutational data further supported the biological relevance of the identified gene set, with concordance observed between mutation-based stratification and transcriptomic grouping, both of which aligned with the established ovarian cancer subtypes reported in prior literature. The co-expression network constructed from the final features revealed four coherent molecular groups among the seven ovarian cancer cell lines. Group 1 (COV362.4, OVCAR-5, OVCAR-8) reflected high-grade serous-like features enriched for metabolic and ribosomal genes, while Group 2 (SKOV3, TOV21G) displayed signatures consistent with clear cell and endometrioid biology. OVCAR-3 (Group 3) emerged as a distinct chemoresistant serous model enriched for extracellular matrix remodeling and inflammatory pathways, whereas IGROV-1 (Group 4) exhibited a hybrid profile bridging serous and endometrioid lineages. Importantly, the inclusion of mutation information validated these subgroupings, providing additional evidence for the robustness of the classification. Together, these findings demonstrate that combining unsupervised and supervised feature selection with network-based modeling provides a powerful strategy for reducing dimensionality, identifying subtype-specific transcriptional programs, and validating molecular stratification in ovarian cancer cell lines. This approach not only advances our understanding of ovarian cancer heterogeneity, but also lays the groundwork for improved model selection in preclinical studies and for refining subtype-specific therapeutic strategies.

## Figures and Tables

**Table 1:** Sample correlation matrix of features X1–X6. The table presents pairwise Pearson correlation coefficients between six features (X1–X6). Each cell represents the strength and direction of the linear association between two features, with values ranging from −1 (perfect negative correlation) to +1 (perfect positive correlation). The diagonal elements are all equal to 1.0, reflecting the perfect self-correlation of each feature. For example, the correlation between X1 and X2 is 0.8, indicating a strong positive linear relationship.

	X1	X2	X3	X4	X5	X6
**X1**	1.0	0.8	0.9	0.4	0.6	0.3
**X2**	0.8	1.0	0.7	0.5	0.4	0.8
**X3**	0.9	0.7	1.0	0.2	0.6	0.4
**X4**	0.4	0.5	0.2	1.0	0.9	0.8
**X5**	0.6	0.4	0.6	0.9	1.0	0.7
**X6**	0.3	0.8	0.4	0.8	0.7	1.0

**Table 2:** Significance matrix of features X1–X6 after applying correlation thresholding. The table shows a binary significance matrix derived from the correlation matrix of features X1–X6, where a threshold of T = 0.7 was applied. Each cell is assigned a value of 1 if the Pearson correlation between two features exceeds the threshold, and 0 otherwise. The diagonal elements are 0 by definition, as self-comparisons were excluded. For example, the correlation between X1 and X2 was 0.8 in the correlation matrix, which exceeded the threshold of 0.7, and therefore marked as 1 in this matrix.

	X1	X2	X3	X4	X5	X6
**X1**	0	1	1	0	0	0
**X2**	1	0	1	0	0	0
**X3**	1	1	0	0	0	0
**X4**	0	0	0	0	1	1
**X5**	0	0	0	1	0	1
**X6**	0	1	0	1	1	0

**Table 3:** Training performance of the classification models. The table summarizes the performance of multiple classifiers during training, evaluated using accuracy, sensitivity, specificity, F1 score, and AUC. Both logistic regression and random forest achieved the highest performance across all metrics, demonstrating perfect classification with no observed variance. Best performing models are marked in bolded text.

Model	Accuracy	Sensitivity	Specificity	F1 Score	AUC
Logistic regression	**1.00 ± 0.00** **(1.00, 1.00)**	**1.00 ± 0.00** **(1.00, 1.00)**	**1.00 ± 0.00** **(1.00, 1.00)**	**1.00 ± 0.00** **(1.00, 1.00)**	**1.00 ± 0.00** **(1.00, 1.00)**
Random forest	**1.00 ± 0.00** **(1.00, 1.00)**	**1.00 ± 0.00** **(1.00, 1.00)**	**1.00 ± 0.00** **(1.00, 1.00)**	**1.00 ± 0.00** **(1.00, 1.00)**	**1.00 ± 0.00** **(1.00, 1.00)**
XGBoost	0.86 ± 0.20 (0.68, 1.00)	0.81 ± 0.32 (0.53, 1.00)	0.90 ± 0.22 (0.70, 1.00)	0.84 ± 0.26 (0.61, 1.00)	0.86 ± 0.20 (0.68, 1.00)
AdaBoost	0.97 ± 0.06 (0.92, 1.00)	1.00 ± 0.00 (1.00, 1.00)	0.90 ± 0.22 (0.70, 1.00)	0.98 ± 0.04 (0.95, 1.00)	0.95 ± 0.11 (0.85, 1.00)
Decision tree	0.91 ± 0.13 (0.80, 1.00)	0.92 ± 0.18 (0.76, 1.00)	0.93 ± 0.15 (0.80, 1.00)	0.93 ± 0.11 (0.83, 1.00)	0.93 ± 0.10 (0.84, 1.00)
SVM	0.91 ± 0.13 (0.80, 1.00)	1.00 ± 0.00 (1.00, 1.00)	0.77 ± 0.32 (0.48, 1.00)	0.94 ± 0.09 (0.86, 1.00)	0.98 ± 0.04 (0.95, 1.00)

**Table 4:** Evaluation performance of the classification models. The table presents the model evaluation results based on accuracy, sensitivity, specificity, F1 score, and AUC. Both logistic regression and random forest maintained the highest performance during evaluation, achieving perfect classification across all metrics. Best performing models are marked in bolded text.

Model	Accuracy	Sensitivity	Specificity	F1 Score	AUC
Logistic regression	**1.00 ± 0.00** **(1.00, 1.00)**	**1.00 ± 0.00** **(1.00, 1.00)**	**1.00 ± 0.00** **(1.00, 1.00)**	**1.00 ± 0.00** **(1.00, 1.00)**	**1.00 ± 0.00** **(1.00, 1.00)**
Random forest	**1.00 ± 0.00** **(1.00, 1.00)**	**1.00 ± 0.00** **(1.00, 1.00)**	**1.00 ± 0.00** **(1.00, 1.00)**	**1.00 ± 0.00** **(1.00, 1.00)**	**1.00 ± 0.00** **(1.00, 1.00)**
XGBoost	0.91 ± 0.13 (0.80, 1.00)	0.86 ± 0.22 (0.67, 1.00)	1.00 ± 0.00 (1.00, 1.00)	0.91 ± 0.14 (0.78, 1.00)	0.93 ± 0.11 (0.83, 1.00)
AdaBoost	0.97 ± 0.06 (0.92, 1.00)	1.00 ± 0.00 (1.00, 1.00)	0.90 ± 0.22 (0.70, 1.00)	0.98 ± 0.04 (0.95, 1.00)	0.95 ± 0.11 (0.85, 1.00)
Decision tree	0.97 ± 0.06 (0.92, 1.00)	1.00 ± 0.00 (1.00, 1.00)	0.93 ± 0.15 (0.80, 1.00)	0.98 ± 0.05 (0.93, 1.00)	0.97 ± 0.07 (0.90, 1.00)
SVM	0.83 ± 0.12 (0.72, 0.93)	1.00 ± 0.00 (1.00, 1.00)	0.60 ± 0.28 (0.36, 0.84)	0.88 ± 0.08 (0.80, 0.95)	1.00 ± 0.00 (1.00, 1.00)

## Data Availability

Data will be available on request.

## Supplementary Materials

The following supporting information can be downloaded at: https://www.mdpi.com/article/10.3390/cancers17213509/s1, Figure S1: Bubble chart of significant pathways identified through enrichment analysis. The x-axis represents enriched biological pathways, and the y-axis shows the corresponding p-values, with lower values indicating higher statistical significance. Bubble size reflects the relative number of genes associated with each pathway, while bubble color indicates the pathway source database. This visualization highlights key functional categories and molecular processes implicated in the dataset, with several pathways reaching strong significance (*p* < 0.05). References [78,79,80,81,82,83,84,85,86,87,88,89,90] are cited in the Supplementary Materials.
