# Supplementary material for: Subtype Characterization of Ovarian Cancer Cell Lines Using Machine Learning and Network Analysis: A Pilot Study
Source: Cancers (Basel). 2025 Oct 31;17(21):3509. doi: 10.3390/cancers17213509 (PMC12607470; doi:10.3390/cancers17213509)
Supplement: Supplementary file 1 [file cancers-17-03509-s001.zip › cancers-3939057-supplementary.pdf]

Pathway Analysis

To further interpret the biological significance of the identified mRNA biomarkers, pathway enrichment analysis was performed using multiple curated resources including GO:BP, GO:MF, GO:CC, KEGG, Reactome (REAC), WikiPathways (WP), MSigDB, and PANTHER. The analysis revealed several enriched pathways and functional modules relevant to ovarian cancer biology, spanning DNA repair, signaling, immune regulation, and cellular metabolism.

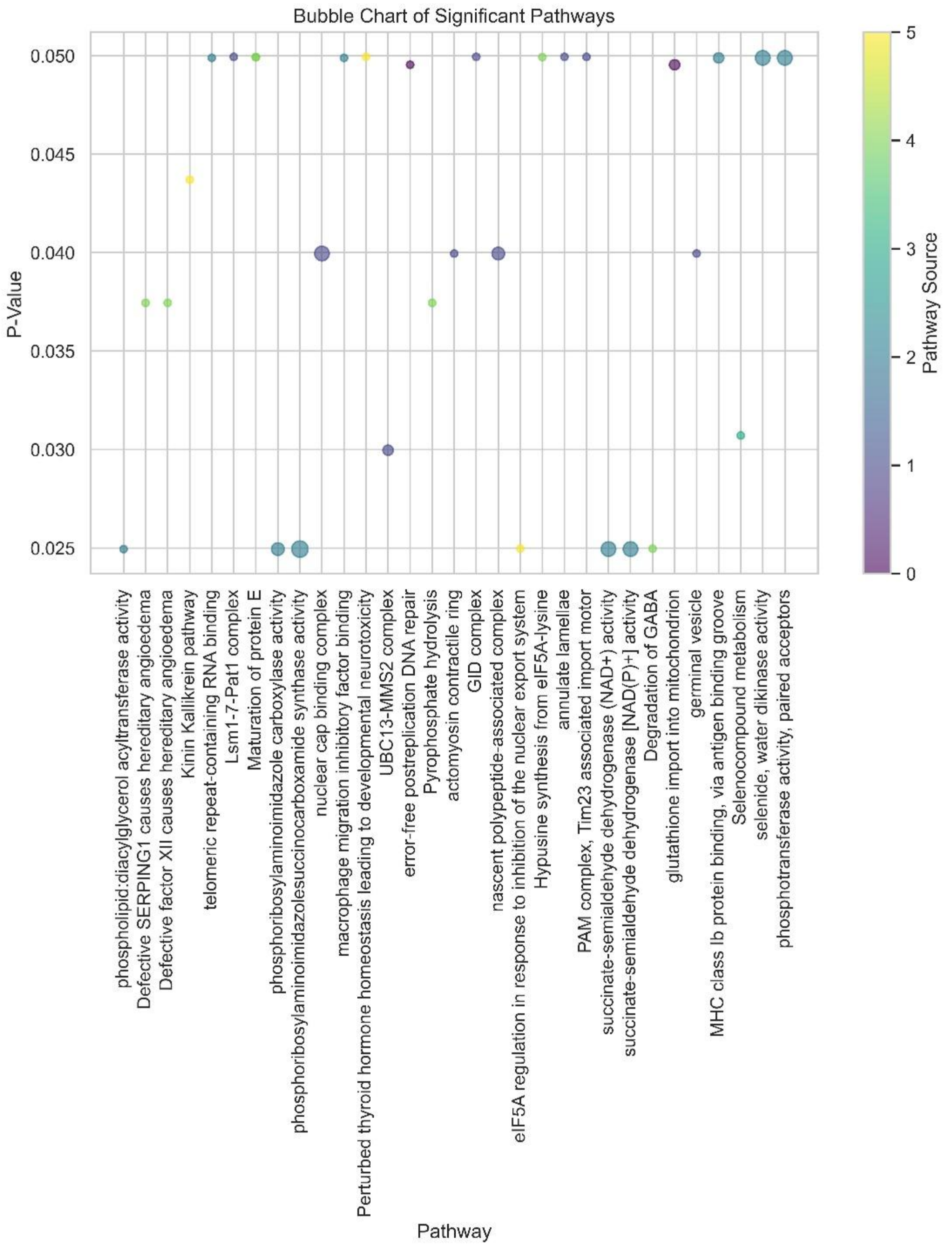

Figure S1. Bubble chart of significant pathways identified through enrichment analysis. The x-axis represents enriched biological pathways, and the y-axis shows the corresponding p-values, with lower values indicating higher statistical significance. Bubble size reflects the relative number of genes associated with each pathway, while bubble color

indicates the pathway source database. This visualization highlights key functional categories and molecular processes implicated in the dataset, with several pathways reaching strong significance ($p < 0.05$).

1. DNA Damage Response and Repair Pathways

Significant enrichment was observed in pathways related to error-free postreplication DNA repair (GO:0042275) and telomeric repeat-containing RNA binding (GO:0061752). These results are consistent with the well-documented genomic instability and defective DNA repair mechanisms that characterize high-grade serous ovarian cancer (HGSOC) (The Cancer Genome Atlas Research Network [1,2]. The involvement of nuclear complexes such as the UBC13-MMS2 complex (GO:0031372) and nuclear cap binding complex (GO:0005846) further underscores the role of mRNA processing and post-transcriptional regulation in maintaining genomic integrity [3].

2. Protein Maturation and Translational Control

The enrichment of maturation of protein E (REAC:R-HSA-9683683, R-HSA-9694493) and complexes such as the nascent polypeptide-associated complex (GO:0005854) and eIF5A regulation in response to inhibition of eIF4A (WP:WP3302) highlights disruption in protein folding and translational control. Dysregulation of translational machinery has been linked to uncontrolled proliferation and chemoresistance in ovarian cancer [4,5]. Additionally, enrichment of hypusine synthesis from eIF5A-lysine (REAC:R-HSA-204626) suggests alterations in translation initiation, a crucial step in tumor cell survival [6].

3. Immune and Inflammatory Pathways

Enrichment was also observed in the kinin-kallikrein pathway (WP:WP5089) and defective complement regulators such as SERPING1 and factor XII (REAC:R-HSA-9657689, R-HSA-9657688). These pathways are central to inflammation and angiogenesis, which tumors exploit for growth and metastasis [7,8]. Additional enrichment in macrophage migration inhibitory factor (MIF) binding (GO:0035718) and MHC class Ib protein binding (GO:0023030) points to modulation of the immune microenvironment, increasingly recognized as a determinant of ovarian cancer progression and treatment response [9,10].

4. Cellular Structures and Complexes

Enriched pathways such as the actomyosin contractile ring (GO:0005826), annulate lamellae (GO:0005642), and germinal vesicle (GO:0042585) reflect alterations in cytoskeletal dynamics and nuclear envelope organization, both critical for cell division, migration, and genomic stability [11,12]. Dysregulation of these structures has been reported in aggressive ovarian cancer, where aberrant cytoskeletal remodeling supports invasion and metastasis [13].

5. Metabolic Reprogramming

The pathway analysis also highlighted selenocompound metabolism (KEGG:00450), succinate-semialdehyde dehydrogenase activity (GO:0004777, GO:0009013), and glutathione import into mitochondria (GO:0160007). These findings are indicative of metabolic rewiring, enabling tumor cells to

sustain rapid growth and adapt to oxidative stress. Glutathione metabolism, in particular, has been strongly linked to chemoresistance in ovarian cancer [6,14].

Overall, the pathway enrichment analysis reveals that the mRNA biomarkers selected by the hybrid feature selection method converge on biologically relevant processes for ovarian cancer, including DNA repair, translational control, immune regulation, cytoskeletal remodeling, and metabolic reprogramming. These results not only validate the biomarker selection approach but also provide mechanistic insights into ovarian cancer heterogeneity. Importantly, several of these pathways (e.g., DNA repair, glutathione metabolism, immune modulation) represent therapeutically actionable targets, supporting the translational potential of this integrative framework for biomarker discovery and personalized treatment strategies.

References

1. Cancer Genome Atlas Research Network. (2011). Integrated genomic analyses of ovarian carcinoma. *Nature*, *474*(7353), 609.
2. Lord, C. J., & Ashworth, A. (2016). BRCAness revisited. *Nature Reviews Cancer*, *16*(2), 110-120.
3. Chen, J., & Weiss, W. A. (2015). Alternative splicing in cancer: implications for biology and therapy. *Oncogene*, *34*(1), 1-14.
4. Bhat, M., Robichaud, N., Hulea, L., Sonenberg, N., Pelletier, J., & Topisirovic, I. (2015). Targeting the translation machinery in cancer. *Nature reviews Drug discovery*, *14*(4), 261-278.
5. Ascenzi, P., Di Masi, A., Sciorati, C., & Clementi, E. (2010). Peroxynitrite—An ugly biofactor?. *Biofactors*, *36*(4), 264-273.
6. Ortega, A. L., Mena, S., & Estrela, J. M. (2011). Glutathione in cancer cell death. *Cancers*, *3*(1), 1285-1310.
7. Hanahan, D., & Weinberg, R. A. (2011). Hallmarks of cancer: the next generation. *cell*, *144*(5), 646-674.
8. Ricklin, D., Hajishengallis, G., Yang, K., & Lambris, J. D. (2010). Complement: a key system for immune surveillance and homeostasis. *Nature immunology*, *11*(9), 785-797.
9. Leng, L., Metz, C. N., Fang, Y., Xu, J., Donnelly, S., Baugh, J., ... & Bucala, R. (2003). MIF signal transduction initiated by binding to CD74. *The Journal of experimental medicine*, *197*(11), 1467-1476.
10. Hamanishi, J., Mandai, M., Ikeda, T., Minami, M., Kawaguchi, A., Murayama, T., ... & Konishi, I. (2015). Safety and antitumor activity of anti–PD-1 antibody, nivolumab, in patients with platinum-resistant ovarian cancer. *Journal of Clinical Oncology*, *33*(34), 4015-4022.
11. Banerjee, S., & Kaye, S. B. (2013). New strategies in the treatment of ovarian cancer: current clinical perspectives and future potential. *Clinical cancer research*, *19*(5), 961-968.
12. Ridley, A. J. (2001). Rho GTPases and cell migration. *Journal of cell science*, *114*(15), 2713-2722.
13. Worzfeld, T., Pogge von Strandmann, E., Huber, M., Adhikary, T., Wagner, U., Reinartz, S., & Müller, R. (2017). The unique molecular and cellular microenvironment of ovarian cancer. *Frontiers in oncology*, *7*, 24.

14. Godwin, A. K., Meister, A., O'Dwyer, P. J., Huang, C. S., Hamilton, T. C., & Anderson, M. E. (1992). High resistance to cisplatin in human ovarian cancer cell lines is associated with marked increase of glutathione synthesis. *Proceedings of the National Academy of Sciences*, *89*(7), 3070-3074.
